# Supplementary material for: Comorbidity and health-related quality of life in people with a chronic medical condition in randomised clinical trials: An individual participant data meta-analysis
Source: PLoS Med. 2023 Jan 17;20(1):e1004154. doi: 10.1371/journal.pmed.1004154 (PMC9844862; doi:10.1371/journal.pmed.1004154)
Supplement: S1 Modelling description — (DOCX) [file pmed.1004154.s002.docx]

Supplementary Appendix: S1_Modelling_Description

# Detailed description of modelling

For each of the following 3 analyses, we first fit individual trial level models within each trial repository. Using the results from all the trial-specific models we then performed meta-analyses.

## i) Association between comorbidity count and quality of life at baseline

### Individual-trial level model

For each trial, we fitted the following linear models within each trial repository.

$${base}_{i}=\beta_{0}+\beta_{1}\cdot age_{i}+\beta_{2}\cdot sex_{i}+\beta_{3}\cdot comorbidity_{i}$$

Where ${base}_{i}$ was the quality of life score at baseline and the $\beta$ parameters 0, 1, 2 and 3 were the coefficients for the intercept, age, sex and comorbidity count respectively.$i$ indicates the individual within each trial. A model was fitted separately for each of the $j$ trials (subscript not shown above for clarity). For each trial, we exported the resultant model coefficients ($\beta_{0}$, $\beta_{1}$, $\beta_{2}$, $\beta_{3}$) standard errors ($se_{\beta_{0}}$, $se_{\beta_{1}}$, $se_{\beta_{2}}$, $se_{\beta_{3}}$ ), and associated variance-covariance matrices for subsequent meta-analysis. The variance-covariance matrices had been exported in case more complex meta-analysis models needed to be fitted (with a multivariate normal likelihood). However, as there was no evidence of departure from linearity only a single coefficient was meta-analysed for each trial in any given model, hence these were not used in any of the subsequent analysis. The matrices are available on our github repository in case they are useful for other researchers.

### Meta-analysis model

The meta-analysis model had a normal likelihood and linear predictor as follows:-

$$\beta_{3,j}\sim N\left( \theta_{j},se_{beta_{3,j}} \right)$$

$$\theta_{j}=\alpha+trial_{j}+cond_{k}+{compar}_{l}$$

$\alpha$ indicates the overall effect. Trial, cond and compar indicate the trial-level, condition-level and treatment comparison-level random effects for the j-trials, k-conditions and l- treatment comparisons. Note that trials were nested within treatment comparisons and conditions, but conditions and treatment comparisons were cross-classified.

The variation in the comorbidity-quality of life associations at the trial, condition and treatment comparison effects was assumed to be normally distributed (random effects):-

$$trial_{j}\sim N\left( \mu_{trial},\sigma_{trial} \right)$$

$$cond_{k}\sim N\left( \mu_{cond},\sigma_{cond} \right)$$

$${compar}_{l}\sim N\left( \mu_{compar},\sigma_{compar} \right)$$

The prior for the overall intercept $\alpha$ was t-distributed with 3 degrees of freedom:-

$$\alpha\sim student\text{-}t\left( df=3,mean=0,sd=10 \right)$$

For the trial, condition and treatment comparison-level random effects, the priors for mean and standard deviation were t-distributed and half-t distributed respectively:-

$$\mu_{trial}\sim student\text{-}t\left( df=3,mean=0,sd=1 \right)$$

$$\mu_{cond}\sim student\text{-}t\left( df=3,mean=0,sd=1 \right)$$

$$\mu_{compar}\sim student\text{-}t\left( df=3,mean=0,sd=1 \right)$$

$$\sigma_{trial}\sim half\text{-}student\text{-}t\left( df=3,mean=0,sd=1 \right)$$

$$\sigma_{cond}\sim half\text{-}student\text{-}t\left( df=3,mean=0,sd=1 \right)$$

$$\sigma_{compar}\sim half\text{-}student\text{-}t\left( df=3,mean=0,sd=1 \right)$$

α was the overall estimate for the association across all treatment comparisons and conditions. The condition-level and treatment comparison-level odds ratios were estimated as follows using samples from the posterior:-

$$effect_{k}=\alpha+cond_{k}$$

$$effect_{l}=\alpha+{compar}_{l}$$

For all parameters the mean of the distribution was obtained as the point estimate. The credible intervals were obtained as the 2.5th and 97.5th percentiles.

## ii) Association between comorbidity count and change in quality of life at trial follow up

### Individual-trial level model

$${final}_{i}=\mathrm{base}_{i}*\gamma+ \beta_{0}+\beta_{1}\cdot age_{i}+\beta_{2}\cdot sex_{i}+\beta_{3}\cdot comorbidity_{i}$$

Final refers to the final observed quality of life score and γ to the coefficient for the quality of life score at baseline. The modelling of final score conditional on the score at baseline means that this can be described as an ANCOVA model.

## Meta-analysis model

As per meta-analysis model (i).

### Departure from linearity

For this analysis we also looked for evidence of departure from linearity in the comorbidity count/quality of life association by adding a comorbidity squared term to the trial-level model and meta-analysing the estimate for β4 rather than β3:-

$${final}_{i}={\gamma\cdot base_{i}+ \beta}_{0}+\beta_{1}\cdot age_{i}+\beta_{2}\cdot sex_{i}+\beta_{3}\cdot comorbidity_{i}+\beta_{4}\cdot\mathrm{comorbiditycount}_{i}^{2}$$

$$\beta_{4,j}\sim N\left( \theta_{j},se_{beta_{4,j}} \right)$$

## iii) Association between comorbidity count and the effect of treatment on change in quality of life at trial follow up

### Individual-trial level model

$${final}_{i}=\mathrm{base}_{i}*\gamma+ \beta_{0}+\beta_{1}\cdot age_{i}+\beta_{2}\cdot sex_{i}+\beta_{3}\cdot comorbidity_{i}+$$

$$\delta_{1}\cdot arm_{i} +\delta_{2}\cdot arm_{i}\cdot comorbidity_{i}$$

Arm refers to the treatment arm, δ1 and δ2 refer to the treatment-arm effect and treatment-comorbidity interaction effect respectively.

### Meta-analysis model

$$\delta_{2,j}\sim N\left( \theta_{j},se_{\delta_{2,j}} \right)$$

Remaining description as per meta-analysis model (i).

### Samples from the posterior

In order to allow other researchers to use the treatment-covariate interaction results to inform subsequent analyses (eg as an informative prior) we also obtained samples from the posterior. We summed 1,000 draws from $\alpha, \mu_{compar}, {\mathrm{and} \mu}_{cond}$to obtain 1000 predictions (y) for each treatment comparison/index condition included in the modelling and for a notional treatment comparison and index condition not included in the modelling. For the latter $\mu_{compar}, {\mathrm{and} \mu}_{cond}$for each sample was randomly drawn from one of the existing levels. Note that μ_trial_  was not included as only the estimates at the level of treatment comparison and index condition were of interest.

We then fitted a t-distribution to y.

$$y \sim student-t(df=d, mean= m, sd=s)$$

d had a gamma (shape = 2, rate = 0.1) prior, m a student-t prior (df = 3, mean = 0, s = 2.5) and s a half-student-t prior (df = 3, mean = 0, s = 2.5). We compared the fit of these distributions to the samples graphically. These plots and the point estimates for d, m and s are available at the project github repository (Outputs/summarise_posteriors.csv and Outputs/summarise_prior_t_distribution.pdf respectively) for use as informative priors.

## iv) Selection of priors

In line with the recommendations for prior collections in the Stan guide (<https://github.com/stan-dev/stan/wiki/Prior-Choice-Recommendations>), we chose “weakly informative” priors for the overall intercepts (SD =10) but only “generally weakly informative” priors (SD =1) for the between trial, between treatment comparison and between condition parameters. Nonetheless, as there were a relatively large number of trials, treatment comparisons and conditions in our data, we also re-fit the models using a "weakly informative" priors for all 3 (between trial, between treatment comparison and between condition). On doing so, we found the results were very similar to the main analysis. (for a table comparing the results under both types of prior please see [https://github.com/ChronicDiseaseEpi/como_qol_public/blob/main/Outputs/compare_weak_generic_priors.csv](https://github.com/ChronicDiseaseEpi/como_qol/blob/main/Outputs/compare_weak_generic_priors.csv)).
