# Supplementary material for: Comorbidity and health-related quality of life in people with a chronic medical condition in randomised clinical trials: An individual participant data meta-analysis
Source: PLoS Med. 2023 Jan 17;20(1):e1004154. doi: 10.1371/journal.pmed.1004154 (PMC9844862; doi:10.1371/journal.pmed.1004154)
Supplement: S1 Additional figures tables — (DOCX) [file pmed.1004154.s003.docx]

Supplementary Appendix:

S1_Additional_Figures_Tables

# Figure A: Comparison of magnitude of the association between comorbidity and quality of life and the effect of treatment on quality of life


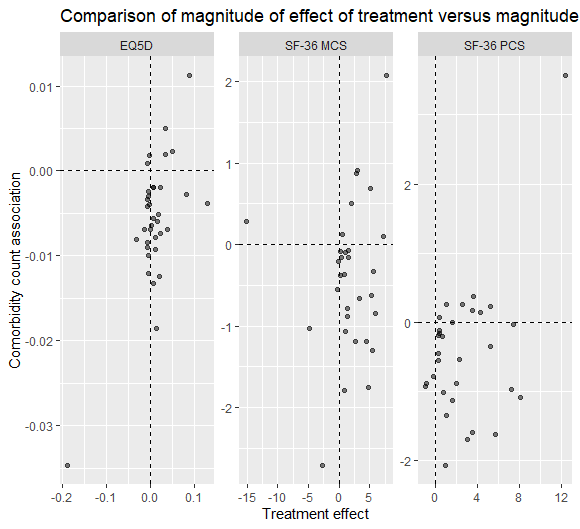


Each data point is a single trial taken from a model of final quality of life score on baseline quality of life score, age, sex, treatment arm, comorbidity treatment interaction, treatment arm and comorbidity count with the latter two coefficients plotted on the x and y axis respectively.

# Table A: Clinicaltrials.gov identifiers, index condition and intervention drug class of included trials

| **nct_id** | **Index_condition** | **Drug_class** |
| --- | --- | --- |
| NCT01209702 | Axial Spondyloarthritis | L04AC |
| NCT00265083 | Axial Spondyloarthritis | L04AB |
| NCT01264939 | Chronic idiopathic urticaria | R03DX |
| NCT01287117 | Chronic idiopathic urticaria | R03DX |
| NCT01292473 | Chronic idiopathic urticaria | R03DX |
| NCT00348140 | Dementia | A10BG |
| NCT00348309 | Dementia | A10BG |
| NCT00428090 | Dementia | A10BG |
| NCT00734474 | Diabetes Mellitus, Type 2 | A10BJ |
| NCT01064687 | Diabetes Mellitus, Type 2 | A10BJ |
| NCT01075282 | Diabetes Mellitus, Type 2 | A10BJ |
| NCT01164501 | Diabetes Mellitus, Type 2 | A10BK |
| NCT01191268 | Diabetes Mellitus, Type 2 | A10BJ |
| NCT01624259 | Diabetes Mellitus, Type 2 | A10BJ |
| NCT01719003 | Diabetes Mellitus, Type 2 | A10BK |
| NCT01106625 | Diabetes Mellitus, Type 2 | A10BK |
| NCT01106677 | Diabetes Mellitus, Type 2 | A10BK |
| NCT01137812 | Diabetes Mellitus, Type 2 | A10BK |
| NCT00783718 | Inflammatory Bowel Disease | L04AA |
| NCT00487539 | Inflammatory Bowel Disease | L04AB |
| NCT00488631 | Inflammatory Bowel Disease | L04AB |
| NCT01224171 | Inflammatory Bowel Disease | L04AA |
| NCT00207766 | Inflammatory Bowel Disease | L04AB |
| NCT01369329 | Inflammatory Bowel Disease | L04AC |
| NCT01369342 | Inflammatory Bowel Disease | L04AC |
| NCT00231595 | Migraine Disorders | N03AX |
| NCT00046254 | Osteoporosis | M05BA |
| NCT00100620 | Osteoporosis | M05BA |
| NCT00439647 | Osteoporosis | M05BA |
| NCT00670501 | Osteoporosis | H05AA |
| NCT00466167 | Parkinson Disease | N04BC |
| NCT01646177 | Psoriasis | L04AC |
| NCT01358578 | Psoriasis | L04AC |
| NCT01365455 | Psoriasis | L04AC |
| NCT00267969 | Psoriasis | L04AC |
| NCT00265096 | Psoriatic Arthritis | L04AB |
| NCT01009086 | Psoriatic Arthritis | L04AC |
| NCT01077362 | Psoriatic Arthritis | L04AC |
| NCT01335464 | Pulmonary Fibrosis | L01XE |
| NCT01335477 | Pulmonary Fibrosis | L01XE |
| NCT00125918 | Pulmonary Hypertension | G04BE |
| NCT00472199 | Restless Legs Syndrome | N04BC |
| NCT00106535 | Rheumatoid Arthritis | L04AC |
| NCT01007435 | Rheumatoid Arthritis | L04AC |
| NCT01119859 | Rheumatoid Arthritis | L04AC |
| NCT01232569 | Rheumatoid Arthritis | L04AC |
| NCT00236028 | Rheumatoid Arthritis | L04AB |
| NCT00264537 | Rheumatoid Arthritis | L04AB |
| NCT00264550 | Rheumatoid Arthritis | L04AB |
| NCT00361335 | Rheumatoid Arthritis | L04AB |
| NCT00973479 | Rheumatoid Arthritis | L04AB |
| NCT00410384 | Systemic Lupus Erythematosus | L04AA |
| NCT00424476 | Systemic Lupus Erythematosus | L04AA |
| NCT00291330 | Venous Thromboembolism | B01AE |
| NCT00558259 | Venous Thromboembolism | B01AE |
| NCT00680186 | Venous Thromboembolism | B01AE |

# Table B: Association of comorbidity count squared and quality of life measures; standardised effect estimates, 95% credibility intervals

| **Model complexity** | **EQ5D Index Value** | **SF36-PCS** | **SF36-MCS** |
| --- | --- | --- | --- |
|  | **35 trials, 24,826 participants** | **31 trials, 14,351 participants** | **31 trials, 14,351 participants** |
| Pooled by Treatment Comparison and Index condition | 0.0008 (-0.0019 to 0.0033) | -0.0014 (-0.0106 to 0.0069) | 0.0004 (-0.0204 to 0.0174); |

# Table C: Summarised posterior predictions (as student t-distributions)

| **Outcome** | **Index condition** | **Drug treatment comparison** | **mean** | **sd** | **df** |
| --- | --- | --- | --- | --- | --- |
| EQ5D | Axial Spondyloarthritis | L04AC | -5.46E-03 | 8.76E-03 | 3.46E+00 |
| EQ5D | Chronic idiopathic urticaria | R03DX | -1.92E-03 | 8.32E-03 | 5.21E+00 |
| EQ5D | Dementia (any) | A10BG | -2.49E-03 | 6.64E-03 | 1.02E+01 |
| EQ5D | Diabetes Mellitus, Type 2 | A10BJ | -3.48E-03 | 6.52E-03 | 1.59E+01 |
| EQ5D | Diabetes Mellitus, Type 2 | A10BK | -2.85E-03 | 6.03E-03 | 9.31E+00 |
| EQ5D | Hypertension, Pulmonary | G04BE | -1.84E-03 | 7.68E-03 | 3.61E+00 |
| EQ5D | Inflammatory bowel disease | L04AB | -8.48E-03 | 9.11E-03 | 4.75E+00 |
| EQ5D | Osteoporosis | H05AA | -1.29E-03 | 6.68E-03 | 5.27E+00 |
| EQ5D | Osteoporosis | M05BA | -2.75E-04 | 5.95E-03 | 2.58E+01 |
| EQ5D | Parkinson's disease (all) | N04BC | -2.04E-03 | 8.14E-03 | 5.60E+00 |
| EQ5D | Psoriasis | L04AC | -7.96E-03 | 9.58E-03 | 4.58E+00 |
| EQ5D | Pulmonary fibrosis | L01XE | 9.16E-04 | 7.28E-03 | 1.23E+01 |
| EQ5D | Rheumatoid arthritis | L04AB | -6.24E-03 | 8.80E-03 | 4.17E+00 |
| EQ5D | Systemic Lupus Erythematosus | L04AA | -5.15E-03 | 7.88E-03 | 7.54E+00 |
| EQ5D | Thromboembolism | B01AE | -2.31E-03 | 5.87E-03 | 2.52E+01 |
| EQ5D | Axial Spondyloarthritis | Unknown | -3.65E-03 | 8.33E-03 | 4.44E+00 |
| EQ5D | Chronic idiopathic urticaria | Unknown | -2.43E-03 | 7.61E-03 | 4.30E+00 |
| EQ5D | Dementia (any) | Unknown | -2.71E-03 | 6.68E-03 | 3.79E+00 |
| EQ5D | Diabetes Mellitus, Type 2 | Unknown | -3.17E-03 | 6.57E-03 | 4.85E+00 |
| EQ5D | Hypertension, Pulmonary | Unknown | -2.02E-03 | 7.93E-03 | 3.68E+00 |
| EQ5D | Inflammatory bowel disease | Unknown | -5.46E-03 | 8.63E-03 | 3.86E+00 |
| EQ5D | Osteoporosis | Unknown | -1.46E-03 | 6.55E-03 | 5.26E+00 |
| EQ5D | Parkinson's disease (all) | Unknown | -2.38E-03 | 7.94E-03 | 4.20E+00 |
| EQ5D | Psoriasis | Unknown | -4.67E-03 | 8.37E-03 | 3.22E+00 |
| EQ5D | Pulmonary fibrosis | Unknown | -9.18E-04 | 7.69E-03 | 5.52E+00 |
| EQ5D | Rheumatoid arthritis | Unknown | -3.53E-03 | 7.65E-03 | 3.36E+00 |
| EQ5D | Systemic Lupus Erythematosus | Unknown | -3.66E-03 | 7.17E-03 | 3.95E+00 |
| EQ5D | Thromboembolism | Unknown | -2.08E-03 | 6.68E-03 | 4.64E+00 |
| EQ5D | Unknown | Unknown | -2.86E-03 | 7.35E-03 | 3.87E+00 |
| SF36-PCS | Axial Spondyloarthritis | L04AB | -5.96E-02 | 5.05E-02 | 6.89E+00 |
| SF36-PCS | Axial Spondyloarthritis | L04AC | -9.84E-03 | 3.46E-02 | 3.65E+00 |
| SF36-PCS | Diabetes Mellitus, Type 2 | A10BK | 1.64E-02 | 2.15E-02 | 2.61E+01 |
| SF36-PCS | Hypertension, Pulmonary | G04BE | -6.34E-03 | 4.14E-02 | 6.66E+00 |
| SF36-PCS | Inflammatory bowel disease | L04AA | -5.87E-03 | 1.98E-02 | 9.96E+00 |
| SF36-PCS | Inflammatory bowel disease | L04AB | -5.94E-02 | 4.43E-02 | 3.59E+01 |
| SF36-PCS | Inflammatory bowel disease | L04AC | -1.01E-02 | 2.89E-02 | 1.22E+01 |
| SF36-PCS | Migraine | N03AX | -1.60E-02 | 3.76E-02 | 6.74E+00 |
| SF36-PCS | Psoriasis | L04AC | -2.14E-03 | 2.78E-02 | 7.58E+00 |
| SF36-PCS | Psoriatic arthropathy | L04AB | -4.74E-02 | 4.59E-02 | 6.89E+00 |
| SF36-PCS | Psoriatic arthropathy | L04AC | 1.23E-03 | 3.18E-02 | 4.26E+00 |
| SF36-PCS | Restless legs syndrome | N04BC | 1.14E-02 | 3.88E-02 | 1.28E+01 |
| SF36-PCS | Rheumatoid arthritis | L04AB | -3.16E-02 | 3.96E-02 | 3.81E+01 |
| SF36-PCS | Rheumatoid arthritis | L04AC | 1.81E-02 | 2.65E-02 | 2.01E+01 |
| SF36-PCS | Systemic Lupus Erythematosus | L04AA | -1.67E-03 | 1.15E-02 | 4.53E+00 |
| SF36-PCS | Axial Spondyloarthritis | Unknown | -1.71E-02 | 3.96E-02 | 3.36E+00 |
| SF36-PCS | Diabetes Mellitus, Type 2 | Unknown | 5.06E-03 | 3.44E-02 | 3.57E+00 |
| SF36-PCS | Hypertension, Pulmonary | Unknown | -6.65E-03 | 3.91E-02 | 4.63E+00 |
| SF36-PCS | Inflammatory bowel disease | Unknown | -1.65E-02 | 3.81E-02 | 6.57E+00 |
| SF36-PCS | Migraine | Unknown | -1.05E-02 | 3.87E-02 | 3.87E+00 |
| SF36-PCS | Psoriasis | Unknown | -8.94E-03 | 3.88E-02 | 5.01E+00 |
| SF36-PCS | Psoriatic arthropathy | Unknown | -7.93E-03 | 3.74E-02 | 3.37E+00 |
| SF36-PCS | Restless legs syndrome | Unknown | 2.97E-05 | 3.79E-02 | 5.21E+00 |
| SF36-PCS | Rheumatoid arthritis | Unknown | 1.08E-02 | 3.26E-02 | 3.88E+00 |
| SF36-PCS | Systemic Lupus Erythematosus | Unknown | -1.05E-02 | 3.18E-02 | 3.69E+00 |
| SF36-PCS | Unknown | Unknown | -4.93E-03 | 3.62E-02 | 3.86E+00 |
| SF36-MCS | Axial Spondyloarthritis | L04AB | -1.59E-02 | 3.73E-02 | 5.92E+00 |
| SF36-MCS | Axial Spondyloarthritis | L04AC | -1.50E-02 | 3.35E-02 | 4.72E+00 |
| SF36-MCS | Diabetes Mellitus, Type 2 | A10BK | -1.03E-02 | 2.18E-02 | 1.21E+01 |
| SF36-MCS | Hypertension, Pulmonary | G04BE | -2.23E-02 | 3.79E-02 | 4.08E+00 |
| SF36-MCS | Inflammatory bowel disease | L04AA | -3.85E-02 | 3.14E-02 | 3.35E+01 |
| SF36-MCS | Inflammatory bowel disease | L04AB | -3.60E-02 | 3.51E-02 | 1.34E+01 |
| SF36-MCS | Inflammatory bowel disease | L04AC | -3.48E-02 | 3.42E-02 | 2.70E+01 |
| SF36-MCS | Migraine | N03AX | -1.78E-02 | 3.74E-02 | 4.93E+00 |
| SF36-MCS | Psoriasis | L04AC | -3.16E-03 | 3.13E-02 | 5.52E+00 |
| SF36-MCS | Psoriatic arthropathy | L04AB | -6.65E-03 | 3.68E-02 | 3.94E+00 |
| SF36-MCS | Psoriatic arthropathy | L04AC | -3.98E-03 | 3.23E-02 | 3.46E+00 |
| SF36-MCS | Restless legs syndrome | N04BC | -5.13E-05 | 3.68E-02 | 5.69E+00 |
| SF36-MCS | Rheumatoid arthritis | L04AB | 3.69E-03 | 2.87E-02 | 7.75E+00 |
| SF36-MCS | Rheumatoid arthritis | L04AC | 6.26E-03 | 2.74E-02 | 1.05E+01 |
| SF36-MCS | Systemic Lupus Erythematosus | L04AA | -7.93E-03 | 1.18E-02 | 2.99E+00 |
| SF36-MCS | Axial Spondyloarthritis | Unknown | -1.57E-02 | 3.28E-02 | 3.29E+00 |
| SF36-MCS | Diabetes Mellitus, Type 2 | Unknown | -1.06E-02 | 2.44E-02 | 3.98E+00 |
| SF36-MCS | Hypertension, Pulmonary | Unknown | -1.78E-02 | 3.45E-02 | 3.91E+00 |
| SF36-MCS | Inflammatory bowel disease | Unknown | -3.49E-02 | 3.35E-02 | 7.19E+00 |
| SF36-MCS | Migraine | Unknown | -1.45E-02 | 2.93E-02 | 3.11E+00 |
| SF36-MCS | Psoriasis | Unknown | -6.61E-03 | 3.40E-02 | 5.13E+00 |
| SF36-MCS | Psoriatic arthropathy | Unknown | -6.00E-03 | 3.67E-02 | 3.51E+00 |
| SF36-MCS | Restless legs syndrome | Unknown | -4.70E-03 | 3.27E-02 | 3.71E+00 |
| SF36-MCS | Rheumatoid arthritis | Unknown | 4.70E-03 | 2.89E-02 | 6.02E+00 |
| SF36-MCS | Systemic Lupus Erythematosus | Unknown | -7.63E-03 | 1.99E-02 | 2.77E+00 |
| SF36-MCS | Unknown | Unknown | -1.37E-02 | 3.00E-02 | 3.49E+00 |

# Table D: Variation in the effect of treatment on quality of life by comorbidity count: sensitivity analysis including only trials demonstrating a benefit in quality of life

| **Model complexity** | **EQ5D Index Value** | **SF36-PCS** | **SF36-MCS** |
| --- | --- | --- | --- |
|  | **12 trials** | **11 trials** | **8 trials** |
| All trials pooled | -0.0137 (-0.0396 to 0.0095) | -0.0065 (-0.1228 to 0.0997) | -0.0503 (-0.2978 to 0.1963) |
| Pooled by drug treatment comparisons | -0.0136 (-0.0491 to 0.0202) | -0.0096 (-0.154 to 0.0955) | -0.0548 (-0.2596 to 0.1472) |
| Pooled by index condition | -0.0152 (-0.0493 to 0.0162) | -0.005 (-0.0776 to 0.0578) | -0.044 (-0.2352 to 0.1516) |
| Pooled by drug treatment comparisons and index condition | -0.0147 (-0.0576 to 0.0255) | -0.0105 (-0.1836 to 0.169) | -0.0418 (-0.3315 to 0.2746) |

# Table E: Search Terms for Existing Literature

| **Pubmed Search Terms** | **Results** |
| --- | --- |
| (comorbidity[Title/Abstract]) AND (multimorbidity[Title/Abstract])) AND (quality of life[Title/Abstract]) | 104 |
| (comorbidity[Title/Abstract]) AND (quality of life[Title/Abstract])) AND (trial[Title/Abstract]) | 302 |
| ("health related quality of life"[Title/Abstract]) AND (comorbidity[Title/Abstract]) | 1624 |
| ("health related quality of life"[Title/Abstract]) AND (multimorbidity[Title/Abstract]) | 221 |
| (("EQ-5D"[Title/Abstract] OR "SF36"[Title/Abstract]) AND ("comorbid"[Title/Abstract] OR "multimorbid"[Title/Abstract])) | 173 |

Search terms reviewed as of Friday 4^th^ March 2022.

# Table F: Summary of included trials by index condition, intervention treatment comparison, outcome measure and comorbidity count

| **Index condition** | **Drug treatment comparisons** |  | **EQ5D** | | | **SF36 (PCS;MCS)** | | |
| --- | --- | --- | --- | --- | --- | --- | --- | --- |
|  |  |  | **Comorbidity Count** | | | **Comorbidity Count** | | |
|  |  |  | **0** | **1** | **≥2** | **0** | **1** | **≥2** |
| Axial Spondyloarthritis | L04AB, L04AC | trials |  |  |  | 2 | 2 | 2 |
| Axial Spondyloarthritis | L04AB, L04AC | n |  |  |  | 157 | 101 | 62 |
| Axial Spondyloarthritis | L04AB, L04AC | age |  |  |  | 37 (10) | 40 (12) | 49 (12) |
| Axial Spondyloarthritis | L04AB, L04AC | male |  |  |  | 77.10% | 67.30% | 64.50% |
| Axial Spondyloarthritis | L04AB, L04AC | base |  |  |  | 45.01 (12.24); 48.53 (13.43) | 43.63 (9.72); 47.36 (13.9) | 37.78 (10.51); 45.72 (15.28) |
| Axial Spondyloarthritis | L04AC | trials | 1 | 1 | 1 |  |  |  |
| Axial Spondyloarthritis | L04AC | n | 58 | 24 | 20 |  |  |  |
| Axial Spondyloarthritis | L04AC | age | 38 (9) | 44 (10) | 54 (13) |  |  |  |
| Axial Spondyloarthritis | L04AC | male | 74.10% | 83.30% | 65% |  |  |  |
| Axial Spondyloarthritis | L04AC | base | 0.77 (0.09) | 0.75 (0.07) | 0.74 (0.08) |  |  |  |
| Chronic idiopathic urticaria | R03DX | trials | 3 | 3 | 3 |  |  |  |
| Chronic idiopathic urticaria | R03DX | n | 122 | 174 | 357 |  |  |  |
| Chronic idiopathic urticaria | R03DX | age | 39 (13) | 43 (13) | 44 (14) |  |  |  |
| Chronic idiopathic urticaria | R03DX | male | 34.40% | 28.20% | 25.80% |  |  |  |
| Chronic idiopathic urticaria | R03DX | base | 0.89 (0.09) | 0.9 (0.08) | 0.88 (0.08) |  |  |  |
| Dementia (any) | A10BG | trials | 3 | 3 | 3 |  |  |  |
| Dementia (any) | A10BG | n | 596 | 677 | 992 |  |  |  |
| Dementia (any) | A10BG | age | 72 (9) | 73 (8) | 75 (7) |  |  |  |
| Dementia (any) | A10BG | male | 40.60% | 42.70% | 40.40% |  |  |  |
| Dementia (any) | A10BG | base | 0.89 (0.09) | 0.88 (0.09) | 0.87 (0.09) |  |  |  |
| Diabetes Mellitus, Type 2 | A10BJ, A10BK | trials | 9 | 9 | 9 |  |  |  |
| Diabetes Mellitus, Type 2 | A10BJ, A10BK | n | 1360 | 1312 | 1774 |  |  |  |
| Diabetes Mellitus, Type 2 | A10BJ, A10BK | age | 57 (11) | 56 (9) | 58 (9) |  |  |  |
| Diabetes Mellitus, Type 2 | A10BJ, A10BK | male | 57.70% | 53.50% | 51.20% |  |  |  |
| Diabetes Mellitus, Type 2 | A10BJ, A10BK | base | 0.94 (0.08) | 0.93 (0.08) | 0.9 (0.09) |  |  |  |
| Diabetes Mellitus, Type 2 | A10BK | trials |  |  |  | 3 | 3 | 3 |
| Diabetes Mellitus, Type 2 | A10BK | n |  |  |  | 412 | 408 | 795 |
| Diabetes Mellitus, Type 2 | A10BK | age |  |  |  | 53 (10) | 56 (9) | 58 (9) |
| Diabetes Mellitus, Type 2 | A10BK | male |  |  |  | 56.10% | 48.80% | 52.10% |
| Diabetes Mellitus, Type 2 | A10BK | base |  |  |  | 58.31 (8.09); 51.72 (7.51) | 55.79 (9.02); 50.65 (7.47) | 53.78 (9.08); 49.72 (7.71) |
| Hypertension, Pulmonary | G04BE | trials | 1 | 1 | 1 | 1 | 1 | 1 |
| Hypertension, Pulmonary | G04BE | n | 36 | 36 | 88 | 36 | 36 | 88 |
| Hypertension, Pulmonary | G04BE | age | 47 (16) | 51 (15) | 57 (14) | 47 (16) | 51 (15) | 57 (14) |
| Hypertension, Pulmonary | G04BE | male | 38.90% | 11.10% | 21.60% | 38.90% | 11.10% | 21.60% |
| Hypertension, Pulmonary | G04BE | base | 0.87 (0.08) | 0.82 (0.08) | 0.82 (0.09) | 49.11 (10.8); 57.39 (11.85) | 45.43 (10.73); 56.36 (13.31) | 43.11 (12.5); 53.23 (13.83) |
| Inflammatory bowel disease | L04AA, L04AB, L04AC | trials |  |  |  | 7 | 7 | 7 |
| Inflammatory bowel disease | L04AA, L04AB, L04AC | n |  |  |  | 1449 | 1141 | 865 |
| Inflammatory bowel disease | L04AA, L04AB, L04AC | age |  |  |  | 36 (12) | 38 (12) | 44 (13) |
| Inflammatory bowel disease | L04AA, L04AB, L04AC | male |  |  |  | 56% | 50.20% | 45% |
| Inflammatory bowel disease | L04AA, L04AB, L04AC | base |  |  |  | 51.33 (11.42); 45.14 (13.13) | 48.98 (10.95); 44.06 (12.55) | 47.82 (11.81); 42.51 (13.1) |
| Inflammatory bowel disease | L04AB | trials | 2 | 2 | 2 |  |  |  |
| Inflammatory bowel disease | L04AB | n | 511 | 377 | 287 |  |  |  |
| Inflammatory bowel disease | L04AB | age | 37 (12) | 39 (13) | 45 (13) |  |  |  |
| Inflammatory bowel disease | L04AB | male | 58.10% | 52.30% | 49.50% |  |  |  |
| Inflammatory bowel disease | L04AB | base | 0.86 (0.09) | 0.85 (0.08) | 0.85 (0.08) |  |  |  |
| Migraine | N03AX | trials |  |  |  | - | 1 | - |
| Migraine | N03AX | n |  |  |  | - | 222 | - |
| Migraine | N03AX | age |  |  |  | - | 40 (11) | - |
| Migraine | N03AX | male |  |  |  | - | 10.80% | - |
| Migraine | N03AX | base |  |  |  | - | 63.48 (12.55); 59.9 (14.06) | - |
| Osteoporosis | H05AA, M05BA | trials | - | 4 | 4 |  |  |  |
| Osteoporosis | H05AA, M05BA | n | - | 1151 | 2639 |  |  |  |
| Osteoporosis | H05AA, M05BA | age | - | 66 (13) | 69 (13) |  |  |  |
| Osteoporosis | H05AA, M05BA | male | - | 47.30% | 39.40% |  |  |  |
| Osteoporosis | H05AA, M05BA | base | - | 0.86 (0.11) | 0.85 (0.1) |  |  |  |
| Osteoporosis | M05BA | trials | 3 | - | - |  |  |  |
| Osteoporosis | M05BA | n | 587 | - | - |  |  |  |
| Osteoporosis | M05BA | age | 67 (10) | - | - |  |  |  |
| Osteoporosis | M05BA | male | 63.50% | - | - |  |  |  |
| Osteoporosis | M05BA | base | 0.88 (0.11) | - | - |  |  |  |
| Parkinson's disease (all) | N04BC | trials | 1 | 1 | 1 |  |  |  |
| Parkinson's disease (all) | N04BC | n | 152 | 109 | 82 |  |  |  |
| Parkinson's disease (all) | N04BC | age | 59 (10) | 61 (9) | 65 (9) |  |  |  |
| Parkinson's disease (all) | N04BC | male | 61.80% | 45.90% | 52.40% |  |  |  |
| Parkinson's disease (all) | N04BC | base | 0.79 (0.1) | 0.76 (0.09) | 0.77 (0.09) |  |  |  |
| Psoriasis | L04AC | trials | 3 | 3 | 3 | 2 | 2 | 2 |
| Psoriasis | L04AC | n | 666 | 269 | 182 | 478 | 275 | 207 |
| Psoriasis | L04AC | age | 43 (13) | 45 (12) | 53 (12) | 43 (12) | 46 (12) | 52 (11) |
| Psoriasis | L04AC | male | 68.50% | 69.10% | 63.20% | 70.30% | 66.20% | 64.70% |
| Psoriasis | L04AC | base | 0.85 (0.13) | 0.83 (0.17) | 0.77 (0.19) | 68.75 (12.31); 64.2 (13.53) | 66.75 (12.68); 64.38 (13.35) | 60.61 (14.86); 61.53 (15.21) |
| Psoriatic arthropathy | L04AB | trials |  |  |  | 1 | - | 1 |
| Psoriatic arthropathy | L04AB | n |  |  |  | 101 | - | 67 |
| Psoriatic arthropathy | L04AB | age |  |  |  | 46 (11) | - | 50 (10) |
| Psoriatic arthropathy | L04AB | male |  |  |  | 62.40% | - | 55.20% |
| Psoriatic arthropathy | L04AB | base |  |  |  | 47.27 (13.09); 53.82 (13.6) | - | 43.9 (13.15); 49.75 (14.88) |
| Psoriatic arthropathy | L04AB, L04AC | trials |  |  |  | - | 3 | - |
| Psoriatic arthropathy | L04AB, L04AC | n |  |  |  | - | 429 | - |
| Psoriatic arthropathy | L04AB, L04AC | age |  |  |  | - | 47 (12) | - |
| Psoriatic arthropathy | L04AB, L04AC | male |  |  |  | - | 53.80% | - |
| Psoriatic arthropathy | L04AB, L04AC | base |  |  |  | - | 42.78 (13.55); 48.55 (16.1) | - |
| Pulmonary fibrosis | L01XE | trials | 2 | 2 | 2 |  |  |  |
| Pulmonary fibrosis | L01XE | n | 197 | 228 | 637 |  |  |  |
| Pulmonary fibrosis | L01XE | age | 64 (8) | 65 (9) | 68 (8) |  |  |  |
| Pulmonary fibrosis | L01XE | male | 87.80% | 85.50% | 74.60% |  |  |  |
| Pulmonary fibrosis | L01XE | base | 0.92 (0.1) | 0.91 (0.09) | 0.88 (0.1) |  |  |  |
| Restless legs syndrome | N04BC | trials |  |  |  | 1 | 1 | 1 |
| Restless legs syndrome | N04BC | n |  |  |  | 81 | 111 | 139 |
| Restless legs syndrome | N04BC | age |  |  |  | 51 (13) | 56 (12) | 61 (10) |
| Restless legs syndrome | N04BC | male |  |  |  | 46.90% | 42.30% | 34.50% |
| Restless legs syndrome | N04BC | base |  |  |  | 67.59 (11.74); 62.01 (12.34) | 63.66 (13.06); 59.75 (15.3) | 61.11 (13.24); 60.9 (12.73) |
| Rheumatoid arthritis | L04AB | trials | 1 | 1 | 1 |  |  |  |
| Rheumatoid arthritis | L04AB | n | 363 | 157 | 71 |  |  |  |
| Rheumatoid arthritis | L04AB | age | 50 (12) | 54 (12) | 58 (11) |  |  |  |
| Rheumatoid arthritis | L04AB | male | 17.60% | 19.70% | 19.70% |  |  |  |
| Rheumatoid arthritis | L04AB | base | 0.73 (0.08) | 0.75 (0.08) | 0.74 (0.07) |  |  |  |
| Rheumatoid arthritis | L04AB, L04AC | trials |  |  |  | 9 | 9 | 9 |
| Rheumatoid arthritis | L04AB, L04AC | n |  |  |  | 1841 | 1442 | 1325 |
| Rheumatoid arthritis | L04AB, L04AC | age |  |  |  | 47 (12) | 52 (12) | 56 (11) |
| Rheumatoid arthritis | L04AB, L04AC | male |  |  |  | 20.90% | 19.80% | 18.10% |
| Rheumatoid arthritis | L04AB, L04AC | base |  |  |  | 44.23 (12.76); 48.96 (14.45) | 41.78 (12.12); 47.91 (14.29) | 40.22 (11.6); 46.47 (14.03) |
| Systemic Lupus Erythematosus | L04AA | trials | 2 | 2 | 2 | 2 | 2 | 2 |
| Systemic Lupus Erythematosus | L04AA | n | 68 | 307 | 737 | 69 | 318 | 739 |
| Systemic Lupus Erythematosus | L04AA | age | 36 (12) | 34 (11) | 40 (12) | 36 (12) | 34 (11) | 40 (12) |
| Systemic Lupus Erythematosus | L04AA | male | 2.90% | 5.20% | 6.40% | 2.90% | 5% | 6.40% |
| Systemic Lupus Erythematosus | L04AA | base | 0.86 (0.09) | 0.86 (0.1) | 0.83 (0.1) | 28.66 (2.55); 21.01 (2.02) | 28.37 (2.4); 21.01 (1.92) | 27.63 (2.49); 20.5 (2.14) |
| Thromboembolism | B01AE | trials | 3 | 3 | 3 |  |  |  |
| Thromboembolism | B01AE | n | 1848 | 1862 | 2740 |  |  |  |
| Thromboembolism | B01AE | age | 50 (15) | 54 (16) | 59 (15) |  |  |  |
| Thromboembolism | B01AE | male | 63.10% | 58.50% | 55.90% |  |  |  |
| Thromboembolism | B01AE | base | 0.88 (0.11) | 0.85 (0.1) | 0.83 (0.1) |  |  |  |
