# Supplementary material for: Comorbidity and health-related quality of life in people with a chronic medical condition in randomised clinical trials: An individual participant data meta-analysis
Source: PLoS Med. 2023 Jan 17;20(1):e1004154. doi: 10.1371/journal.pmed.1004154 (PMC9844862; doi:10.1371/journal.pmed.1004154)
Supplement: Attachment — Submitted filename: response to reviewers.docx [file pmed.1004154.s004.docx]

# Editor’s comments

Please include the code used to determine comorbidity scores

The relevant code is now available within the public github repository for the project here <https://github.com/ChronicDiseaseEpi/como_qol_public>/Supporting

Please report your SR/MA according to the PRISMA guidelines

Done. Please see methods and results sections and attached checklist.

Please provided the completed PRISMA checklist

We have now provided the completed PRISMA checklist.

Please add the following statement, or similar, to the Methods: "This study is reported as per the Preferred Reporting Items for Systematic Reviews and Meta-Analyses (PRISMA) guideline (S1 Checklist)."

Done. Please see page 7 line 127-128.

Your search end dates are not clear

The search dates have been included with the following text: “the United States clinical trial registry at clinicaltrials.gov[33] was searched for eligible studies meeting the following criteria; randomised phase 2/3, 3 or 4 trials studying pre-selected drug classes used to treat or prevent 23 selected long term medical index conditions, registered from January 1990 until the dates of initial IPD access on 21 November 2016 and 18 May 2018, for CSDR and YODA repositories respectively.”

Please see page 7- 8 lines 144-148.

Please update your search to the present time (within the last 6 months, if not already).

Unfortunately, adding any trials to our existing set of IPD trials would be treated as a de novo application by the trial repositories (clinicalstudydatarequest and YODA). This means that adding any trials would result in a delay of at least a year (including the time for steering committee review, sign-off of data sharing agreements, data anonymization by trial sponsors, and IPD data analysis) therefore we do not think that an update would be feasible in this instance.

Please embed abbreviations within the main manuscript text at the time the abbreviation is first referred to and remove from the end of the manuscript.

Done.

Please remove consent/data availability statement/competing interests statement from the end of the manuscript and include in the manuscript submission form instead

Done.

Please report your abstract according to PRISMA for abstracts

Done. Please see pages 3-4.

Please combine the Methods and Findings sections into one section, “Methods and findings”.

Done

Please ensure that all numbers presented in the abstract are present and identical to numbers presented in the main manuscript text.

Done

Please provide the dates of search, data sources, types of study designs included (specify RCT results line 1)

We have now included with the following text: “Included trials were registered on the US trials registry for selected index medical conditions and drug classes, phase 2/3, 3 or 4, had ≥ 300 participants, a non-restrictive upper age limit, and were available on one of two trial repositories on 21/11/2016 and 18/05/2018 respectively.”. Please see page 3 lines 37-39.

Please quantify the main results p values, as well as with 95% CIs.

Done.

Please include the important dependent variables that are adjusted for in the analyses.

We have now added the following text: “Per additional comorbidity, adjusting for age and sex, across all index conditions and treatment comparisons, comorbidity count was associated …”. Please see page 3-4 line 50-51.

In the last sentence of the Abstract Methods and Findings section, please describe the main limitation(s) of the study's methodology.

We have added the following text: “This analysis, limited to trials where IPD was available in two repositories, found no evidence of any interaction between comorbidity count and treatment efficacy for either EQ5D or SF36.” Please see page 4, line 57-59.

At this stage, we ask that you include a short, non-technical Author Summary of your research

Done. Please see pages 4-6, lines 71-100.

Line 69: Please replace “background” with “Introduction”

Done. Please see page 6 line 101.

Please quantify the main results p values, as well as with 95% CIs, where relevant

Done.

Where p-values are reported, please also provide the statistical tests used to determine them

We have expanded the statistical analysis text as follows:-

“Samples from the posterior distribution for each association was summarised as the mean, and the 2.5^th^ and 97.5^th^ percentiles (credible intervals) of the posterior distribution. The proportion of the distribution less than 0 (probability of negative association, i.e., Bayesian P) was also reported” Please see page 11, lines 226-229.

Please report the end date of your search - line 108: “…from January 1990 onwards…”

The following text has been added:” In brief, the United States clinical trial registry at clinicaltrials.gov[33] was searched for eligible studies meeting the following criteria; randomised phase 2/3, 3 or 4 trials studying pre-selected drug classes used to treat or prevent 23 selected long term medical index conditions, registered from January 1990 until the dates of initial IPD access on 21 November 2016 and 18 May 2018, for CSDR and YODA repositories respectively.” Please see page 7-8 line 144-148.

Please evaluate study quality and risk of bias.

As we had access to IPD we were able to make the following assessments of trial robustness to possible biases:- 94.6% were at least double blinded (double blind n=26, triple blind n=8, quadruple blind n= 19), there being only 3 open label trials. Participant completion ranged from 38.4% to 100%, with a mean (sd) completion of 81.1% (13.2%) (median 83.3% and IQR 71.4 – 90.6%). Given this is an IPD analysis there was no reporting bias.

This text has been added to the revised manuscript. Please see page 14 lines 275-279.

Please evaluate evidence of publication bias.

As trials were identified from the US clinical trials register (clinicaltrials.gov) rather than a database of published papers (e.g. PubMed) there was no risk of publication bias. However, this does not mean that the trials were a random or complete sample of all registered trials. As we have noted previously [44]this set of trials were broadly similar to trials where IPD was not available with respect to the set indications, phases, number of participants, start dates and exclusion criteria. However, inflammatory bowel disease and arthritis trials and trials of immunosuppressants were overrepresented while phase 4 trials and those with especially large enrolment sizes were underrepresented.

We have added the above text to the limitation section page 20, lines 380-387.

Please clearly report how you searched for and selected published studies including the search platforms

We have added to the following text: “In brief, the United States clinical trial registry at clinicaltrials.gov[33] was searched for eligible studies meeting the following criteria; randomised phase 2/3, 3 or 4 trials studying pre-selected drug classes used to treat or prevent 23 selected long term medical index conditions, registered from January 1990 until the dates of initial IPD access on 21 November 2016 and 18 May 2018, for CSDR and YODA repositories respectively. Trials were excluded where they examined treatment for neoplastic, infectious, affective, psychotic or developmental disorders. Trials were included if they were registered to clinicaltrials.gov[33] and had IPD availability within Clinical Study Data Request (CSDR) or Yale University Open Data Access (YODA) IPD repositories at the time of the original data request to these platforms. To make efficient use of analyst time (both ours and that of the trial sponsors who anonymised the IPD), included trials were limited to those with ≥300 participants. Given our focus on comorbidity, trials were excluded if they limited participants to those aged less than 60 years old. For the current analysis this set of trials was restricted to those with at least one generic health-related quality of life measure. As our aim in the current analysis was to examine the impact across a range of index conditions, we did not include trials which only included disease specific measures of health-related quality of life (e.g. St George's Respiratory Questionnaire). Included trials were categorised based on index medical condition and the World Health Organization Anatomic Therapeutic Chemical (ATC) drug class of the intervention drug.[34]”

Please see page 7-8 lines 144-160.

Please ensure that your ethics statement is included in the manuscript methods section, as stated in the submission form, and remove from the end of the manuscript

Done. Please see page 12 lines 247-249.

Line 106: “…(Prospero CRD42018048202).(30) The full selection process and analysis plan has been documented previously. (31)” please include the analysis plan with the manuscript, please see also reviewer comments below

The relevant selection process document and statistical analysis plans for CSDR and YODA are now available within the public github repository for the project here :-<https://github.com/ChronicDiseaseEpi/como_qol_public>/Supporting

Please remove the sub-headings from the discussion and place the conclusion as the final paragraph (removing the heading “conclusion”).

Done.

Thank you for including the figure captions and the figures.  Please also place these within the manuscript results section

Done.

I agree with the reviewer comments below that the PRISMA flowchart would be a helpful addition to the main manuscript.

The PRISMA flowchart now appears in the main manuscript as figure 1.

Please consider avoiding the use of red (and green) to make the figures accessible to those with colour blindness

Done.

Table 2 – please also provide unadjusted analyses

These have been added to table 2.

Please select the PLOS Medicine reference style in your citation manager.

Done.

# Reviewer 1

Clearly a lot of work went into doing the first stage of the analysis.

Thank you. We appreciate the recognition of the large amount of work involved in analysing a large number of individual-level participant data trials.

One thing I was curious about was whether you kept any data about how comorbidities were recorded across all of the trials.

Thank-you for this question. We have been able to capture the proportion of people per trial with each comorbidity, based on our definitions, as a summary of comorbidity within the trials. The prevalence of each comorbidity varied between trials. Some trials had levels of certain comorbidities in line with that of a representative community sample used in our previous analysis. Other trials had much lower prevalence of comorbid conditions. This pattern of comorbidity was similar across trials of differing index conditions and no individual comorbidity seemed to be consistently well represented or consistently underrepresented. Please see the supplementary appendix of our previous publication for more detailed information here: <https://static-content.springer.com/esm/art%3A10.1186%2Fs12916-019-1427-1/MediaObjects/12916_2019_1427_MOESM9_ESM.pdf>

Is it possible to please see a copy of the code that derives the comorbidity count?

Yes this is now available at the public github repository here <https://github.com/ChronicDiseaseEpi/como_qol_public/Supporting>

I would consider some adjustment to the title if there is space, in particular to reflect that the study is focused on drug trials of chronic disease patients.

We have adjusted the title to account for this point. Please see page 1 lines 2-3

With only a limited number of data sets available for IPD, do you consider the results to be generalizable to multi-morbidity in all drug RCTs?

We suspect that the observation that comorbidity count at baseline predicts a decline in quality of life is probably straightforwardly generalisable – as this does not depend on specific treatments, and was seen equally in investigational treatment and comparator arms.

We think that the generalisability of the observation that the effect of treatment on quality of life did not differ by comorbidity count is more complex. We think that our findings suggest that assuming no interaction is generally likely to be a reasonable assumption for the kinds of conditions and drug classes covered in our analysis as the main analysis or “base case”. However, if there are plausible biological or clinical reasons why there may difference in treatment efficacy for specific indications, e.g. if a treatment is particularly burdensome, or if there is sufficient data with which to estimate heterogeneity in treatment effects for specific indications (which unfortunately is rarely the case) we would not find it reasonable to make this assumption. We would also suggest that a sensitivity analysis assuming some attenuation of efficacy in people with comorbidity would be advisable.

As such, we have added the following statement to the manuscript:-

“Our findings imply that treatment efficacy estimates for EQ-5D, SF-36-PCS and SF-36-MCS from clinical trials are also likely to be applicable to settings with (at least modestly) higher levels of comorbidity, **at least for the kinds of conditions and drug classes covered in our analysis**” [additions in bold] Please see page 23, lines 448-451.

We have also added a caveat based on the fact that we used comorbidity count to measure multimorbidity, and other operationalisations may yield different conclusions:-

“First although comorbidity must, by definition increase with increases in multimorbidity, a simple count such as we used does not capture the full complexity of multimorbidity, including issues such as interactions between concordant and discordant conditions[43]. It is plausible that more nuanced measures of multimorbidity, feasible in trials designed to collect multimorbidity information prospectively, would lead to more nuanced results as to, for example, the effect of specific combinations of conditions on quality of life and heterogeneity of treatment effects” – Please see page 20, lines 370-376.

One important detail I would add here from the PROSPERO protocol was the disease selection criteria i.e. that ID, cancer etc. are excluded

We have added the following text: “Trials were excluded where they examined treatment for neoplastic, infectious, affective, psychotic or developmental disorders.” Please see page 8 lines 148-149.

To clarify, this means that age was rescaled to be in units of SD?

Yes, the standard deviation of age was approximately 15 years for most of the included trials, therefore results were divided by 15 for all trials to rescale age into a common scale. We have added the following text:-

“Baseline quality of life was modelled adjusting for age (per 15-year increment, which was close to the standard deviation for most trials)”. Please see page 10 lines 209-210.

Were any checks of residuals from this stage done? (I can understand that this would be a pain with so many trials to check). What criteria was used to decide that the polynomial (squared) term of # comorbidities was consistent with linearity?  A second order polynomial is a decent way to account for non-linearity but it won't always work in my experience.

We originally chose simple polynomials as we felt that this would allow us to meta-analyse the data – using trials coming from multiple platforms – more straightforwardly. However, we accept that this does not always capture non-linearity as well as other methods such as penalized regression splines. Therefore, in response to this question, we have now repeated the models for the comorbidity-count association using penalized splines rather than second-order polynomials for comorbidity count, both models gave very similar results. This comparison is available for trials held in the YODA repository, the figure and data are at:-

- <https://github.com/ChronicDiseaseEpi/como_qol_public/Outputs/compare_poly_sq.png>
- <https://github.com/ChronicDiseaseEpi/como_qol_public/Data>.

The sudden jump seen in some panels, and the fact that some standard errors appear to be zero, is due to rounding prior to export from the repository. Due to technical problems within the CSDR repository, this export request has not yet been passed by all the trial sponsors, but the results were similar to those for YODA. We will add these to the public repository when they have been released by CSDR.

I think this is an accurate statement about the main results, one thing I struggled with was translating what the coefficient (i.e. change in HRQOL with one extra comorbidity) translates to. I didn't have a sense of whether this was a very dramatic change or a minor one. I am not sure if this can be changed easily - and might just reflect that I don't usually work with HRQOL outcomes more than any problem with the manuscript.

We also found this aspect challenging during the analysis as we were surprised that we were unable to identify published reports setting out what constitutes a clinically important difference in quality of life measures. In retrospect we wonder if it is because the change in quality of life is just one input to the health economic models used to estimate (for example) incremental cost-effectiveness ratios. To try and provide some context we have reported the trial treatment effect as a benchmark. We would be happy to rescale the reporting to multiples of the mean trial treatment effect if that would be helpful.

This is the posterior probability of a negative interaction beta coefficient?

Thank-you for highlighting that we had not stated this adequately in the methods. We have now added:-

“The proportion of the distribution less than 0 (probability of negative association, i.e. Bayesian P) was also reported.”

Please see page 11 lines 228-229.

I would describe the prior for the overall intercept to be 'weakly informative' (SD=10), but it looks like the trial/cond/comp priors are more strongly informative (SD=1). What was the rationale of this choice (or have I misunderstood this part?)?

Thank-you for raising this issue, we have now added an additional analysis setting the SD to 10 for the trial/cond/comp priors and added a section to the supplementary appendix explaining our prior choices:-

In line with the recommendations for prior collections in the Stan guide (<https://github.com/stan-dev/stan/wiki/Prior-Choice-Recommendations>), we chose “weakly informative” priors for the overall intercepts (SD =10) but only “generally weakly informative” priors (SD =1) for the between trial, between treatment comparison and between condition parameters. Nonetheless, as there were a relatively large number of trials, treatment comparisons and conditi“In line with the recommendations for prior collections in the Stan guide (<https://github.com/stan-dev/stan/wiki/Prior-Choice-Recommendations>), we chose “weakly informative” priors for the overall intercepts (SD =10) but only “generally weakly informative” priors (SD =1) for the between trial, between treatment comparison and between condition parameters. Nonetheless, as there were a relatively large number of trials, treatment comparisons and conditions in our data, we also re-fit the models using a "weakly informative" priors for all 3 (between trial, between treatment comparison and between condition). On doing so, we found the results were very similar to the main analysis. (for a table comparing the results under both types of prior please see <https://github.com/ChronicDiseaseEpi/como_qol_public/blob/main/Outputs/compare_weak_generic_priors.csv>)Please see Supplementary appendix: Selection of priors.

# Reviewer 2

It is a policy relevant piece of research that has potential to contribute to understanding about the causal relationship between multimorbidity and QoL.

Thank you. We are pleased that our research is considered to be relevant to policy.

In the Introduction the study is contextualised in relation to multimorbidity, characterised as two or more conditions. But in the methods the authors pivot to talk about comorbidity and this seems to represent significant conceptual slippage that is not adequately addressed.

Thank-you for raising the issue of the complexity of multimorbidity and the conceptual differences between comorbidity counts and multimorbidity. We have now added the following text to the limitations section:-

“First although comorbidity must, by definition increase with increases in multimorbidity, a simple count such as we used does not capture the full complexity of multimorbidity, including issues such as interactions between concordant and discordant conditions[43]. It is plausible that more nuanced measures of multimorbidity, feasible in trials designed to collect multimorbidity information prospectively, would lead to more nuanced results as to, for example, the effect of specific combinations of conditions on quality of life and heterogeneity of treatment effects”. Please see page 20 lines 370-376.

The number of index conditions (21) seems quite small compared to previous epidemiological studies of multimorbidity

There were 21 conditions included in the comorbidity count rather than 16 (this referred to the number of index medical conditions), although we acknowledge that some previous studies using routinely collected healthcare data have had longer lists of conditions. However, our set of conditions encompasses a range of body systems – including mental health – and could reliably be operationalised using the data available in trial IPD. We are not aware that any previous study has been able to obtain a larger number of comorbidities for a large set of IPD trials.

Excluding trials with populations younger than 60 risks excluding populations with multimorbidity from socio-economically disadvantaged backgrounds where multimorbidity is known to affect people 10 years younger

We apologise for not making it clear that we did not exclude trials with populations younger than 60. Rather we excluded trials that did not allow any participants aged 60 years or older. We did so because we felt that trials which imposed such a low age restriction would likely be particularly restrictive with respect to comorbidities, limiting their usefulness for our planned analyses.

Additionally, the list of index conditions excludes mental health problems, both common and serious (if we exclude dementia as not belonging to that family of clinical health problems). This seems to be a major omission given the prevalence of mental and physical multimorbidity and this should be explained and made clear in the title or at least the main description of the study.

We have clarified in the title and description that the analysis focusses on people with chronic medical conditions. We agree that researching mulitmorbidity within people with chronic mental health problems would be interesting and clinically relevant, however we believed this is beyond the scope of this current work.

# Reviewer 3

I think there is a very important message here that has the potential to impact on important aspects of trial design and interpretation.

Thank you.

My understanding is that the Euroqol group do not consider EQ-5D to be an abbreviation and that this is the full name of the measure and so no need to define this as Euroqol-5 dimension.

Thank you. We have removed the definition from the manuscript.

EQ-5D-3L, EQ-5D-3L, and SF-36 are usually hyphenated in this style

We have now changed to the hyphenated style throughout the manuscript.

This is one of several papers published by this group on this dataset.  I think it would be helpful to briefly document these, and their headline conclusions in the background to make it clear where this paper sits in the overall body of the work

We have added the following text to the introduction: “In previous individual participant data (IPD) analyses from a set of 124 clinical trials ranging across a number of index conditions and treatment comparisons, we have previously shown that multimorbidity, although under-represented is present among trial participants and predicts increased rates of both serious adverse events and trial attrition[5,30,31]. Using the 56 trials from this set for which measures of quality of life are available, we now aim to determine whether comorbidity count, which is increased in multimorbidity, predicts change in quality life and whether treatment effects on quality of life differ by comorbidity count at baseline.”

Please see page 6-7, lines 119-125.

I think there is too much extraneous data in the abstract. Detailing the baseline relationship between co-morbidity & EQ-5D / SF-36 distracts from the main points of interest - that people who are less well do less well overall but that they have just as much to gain from effective treatments. It is the absence of any interaction between co-morbidity and that treatment efficacy that is the golden nugget from this paper. This could be drawn out more in the abstract.

We are pleased that this finding was of interest, and agree from the point of view of treatment decision-making it is possibly the most important observation. However, we are also conscious that other researchers, including some of our co-authors find the observed association between comorbidity count and change in quality of life particularly important (as well as comparatively novel), and have therefore continued to include this observation in the abstract (which has been re-drafted as per the PRISMA abstract guideline). We are of course very willing to compromise on this point if the editorial team felt that this was necessary.

Also, for the more general reader I think some explanation is needed of 'standardised units'

We apologise that this is not clear. We have added the following text to the main manuscript:-

“In order to convert the measures onto a similar scale we standardised each; we did so by dividing the estimates and standard errors by published estimates of the standard deviation (EQ-5D-index – 0.23,[39] SF-36-PCS - 9.08 and SF-36-MCS – 10.16 [40]).”

Please see page 10, lines 213-216.

And to the abstract

“All results are in standardised units (obtained by dividing the EQ5D/SF36 estimates by published population standard deviations).”

Please see page 3, lines 49-50.

I think the full, a priori, statistical analysis plan should be available with this paper.

We have now included the statistical analysis plans from our applications to the IPD repositories.

Thus I assume they are standardising to a UK value set for EQ-5D-3L. If so this should be clear. There should be a link to where this can be found directly rather than the generic Euroqol website.

We apologise for not making this clear. As we had access to UK value sets for both EQ-5D-3L and EQ-5D-5L we did not require to use the Van Hout algorithm to map between the two measures. Instead we used the 3L and 5L value sets respectively to obtain a single index value for each participant. <https://euroqol.org/eq-5d-instruments/valuation-of-eq-5d/>.

We have had the following text:- “Since trials in our analysis included either type, but not both, we mapped each to a single EQ-5D index value (using UK population-based value sets for EQ-5D-3L and EQ-5D-5L respectively to obtain a single value which is comparable across all the EQ-5D trials [37]).”

Please see page 9, lines 182-184.

For this current paper this sentence on lines 155 - 156 is probably superfluous

We have now removed this sentence.

In table 1 do we really mean age down to two places of decimals?  Might whole numbers be easier on the eye and just as informative?  Similarly for percentage of males whole percentages are likely to just as informative as presenting to one decimal place.

We agree and have updated table 1 to reflect this.

In the review copy figures 1&2 were rather blurred.  A clearer version will be needed for publication

Higher resolution versions are now uploaded with the revision.

I think the PRISMA flow chart in the supplementary files might be better included in the main paper

The PRISMA flowchart now appears in the main manuscript as figure 1.
