# Supplementary material for: Comorbidity and health-related quality of life in people with a chronic medical condition in randomised clinical trials: An individual participant data meta-analysis
Source: PLoS Med. 2023 Jan 17;20(1):e1004154. doi: 10.1371/journal.pmed.1004154 (PMC9844862; doi:10.1371/journal.pmed.1004154)
Supplement: Attachment — Submitted filename: response_to_reviewers.docx [file pmed.1004154.s005.docx]

Comments from the academic editor

A couple points in the article that were not entirely clear to me were:
1) how the evidence that they have in the article/appendix about linear relationship between predictor and outcome is consistent with the conclusion that non-linear relationship is not present (as opposed to it is unclear whether the relationship is linear or not) -- the linear assumption may be important if there are some people with outlier # of comorbidities which can have a lot of leverage in a linear model's slope estimates; also trial exclusions may systematically take out some types of comorbidities which could be more influential and hence generalization from trial efficacy and efficacy modification by comorbidity to population effectiveness and effect modification may be more challenging.

We have added the following sentences to address these limitations:-

"Moreover, while there was no evidence of any departure from linearity with the range of comorbidity counts we observed, this may not be true for higher comorbidity counts”

and (new text underlined)

“ As such, care should be taken in extrapolating our findings to individuals with three or more conditions as well as to people with more severe comorbidities who are likely to be excluded from trials since it is possible that these types of comorbidities do modify treatment responses.”

Please see page 20 lines 381-382 and page 20-21 lines 400-403.

2) the baseline QoL in the trials is generally relatively high (almost all 0.80 or above for EQ4D QoL weight) so it is probably important to not overly confidently extrapolate too far out of sample (to patients with baseline QoL that is substantially below) in terms of the impact of comorbidities on change in QoL (e.g., just because comorbidities for relatively healthy people [high QoL at baseline] may not have a very strong impact on the size of a treatment effect does not necessarily imply that this will be the case for people with much lower QoL at baseline [whose QoL is substantially outside the range of the study populations in the trials considered])

We have added the following sentence to the limitations:-

“Moreover, while there was no evidence of any departure from linearity with the range of comorbidity counts we observed, this may not be true for higher comorbidity counts. Similarly, care should be taken when applying our findings to individuals with lower quality of life, since the quality of life scores in the trial participants were generally fairly high at baseline.”

Please see page 20 lines 381-385.

GENERAL
Please address all reviewer and editor comments in full
Please check the in-text reference call-outs throughout the manuscript there is occasionally a space missing between the text and the opening parentheses e.g. line 103 “patients[1]”, line 112 “reported[8–22]” and so on.  Please ensure that punctuation follows closing parentheses e.g. line 282 “…conditions. [30] Trials….” should read "…conditions [30]. Trials….”  Please check and amend throughout.

Apologies for this error. This has now been corrected throughout.

TITLE
suggest “conditions” in place of “diseases” or something similar

We have made this change in the title and throughout as suggested, with the exception of where the word disease is within the recognised condition name e.g. inflammatory bowel disease.

ABSTRACT
Thank you for updating the abstract and reporting according to PRISMA guidance.  We note the comments from reviewer 3 regarding inclusion of information in the abstract and acknowledge that PRISMA reporting necessitates this for purposes of transparent data reporting. In context of the reporting guidance all findings should be reported in the abstract.

To me, not dissimilar to reviewer 3, the finding of an absence of interaction between co-morbidity and treatment efficacy is more interesting and more novel than other findings and I suspect may be to others reading your manuscript also.  I agree with reviewer 3 that with respect to this particular outcome, as written, the importance of this point is somewhat lost, particularly on the general reader.  I would suggest re-wording/elaborating the final sentences of the methods and findings section (lines 57-59) as well as the abstract conclusion such that the meaning/implications of the treatment efficacy data are clearer especially to the more general reader – i.e. what does the negative association mean in real terms at the patient and/or policy level? (see also author summary below)

Thank you. We have now restructured the abstract to address these points. Please see pages 3-4, lines 28-66.

Line 59: “EQ5D or SF36” – should hyphenate here also, please check throughout and amend where necessary.

Apologies, this has been corrected throughout.

AUTHOR SUMMARY
Line 74: perhaps “physical and mental health” instead of “mental function and wellbeing”, or something similar

We have made this change to the author summary. Please see page 5, lines 76-77.

Line 78: beginning “Multimorbidity…” suggest “Multimorbidity, the presence of two or more conditions, makes diagnosis and treatment more complex and is associated with worse quality of life in some settings.” The first sentence, as written, is perhaps redundant?

We have made this change to the author summary. Please see page 5, lines 81-82.

Line 80: beginning “Moreover, people with multimorbidity…” suggest beginning as a separate bulleted point as “People with multimorbidity are….”

We have made this change to the author summary. Please see page 5, lines 83-86.

What do these findings mean?
In general, I think you undersell your study, perhaps in an attempt not to oversell it (which is always important).  I would suggest you revise this section with the below comments in mind:
Line 96: “…which helps inform clinical decision making” – how? Please justify the statement

Line 93: “a higher comorbidity count did not change the effect of treatment on quality of life.” and line 98: “…suggest that the effect of treatments on quality of life scores…” It may help to explicitly/simply state what the effect of treatment is. Throughout this is somewhat left to assumption which may not be helpful to the general reader.

Line 97: “These findings also provide some reassurance for clinicians and guideline developers…” – again how? I would revise this point in line with the above, what is the effect of treatment on QoL scores? I think this could be structured/re-worded to pack a bigger punch – which I think it deserves – it’s important not to overstate findings but I think you’re somewhat understating/underselling the importance of your study outcomes here.

Line 100: “…and for the types of treatments and conditions included in this analysis.” Rather paradoxically the statement is both broad and vague. I appreciate it would be impossible to list these but is there a better way to umbrella the included conditions (or refer to categories of those excluded)?  Perhaps not given the nature of the study, in which case would it be better removed altogether?  I'll leave it to your discretion, but I would have a think about how this is written such that you include as much necessary information as possible.
Thank-you for these recommendations to improve the content and clarity of the section “what do these findings mean”. In response, we have re-written this section as follows:-

- *These findings suggest that where treatments improve quality of life for participants overall, they are similarly likely to do so for people with multimorbidity.*
- *Whether this is true for individuals with higher numbers of comorbidities or with conditions and treatments not included in our analyses remains uncertain.*
- *Nonetheless, our findings help inform clinical decision making by reassuring clinicians, health economists and guideline developers that overall trial results can be used when considering how best to manage many people with multimorbidity*

Please see page 6, lines 97-103.

METHODS and RESULTS
Line 278: “…mean (sd) completion” should this read (SD) if representing ?standard deviation

Apologies, this does refer to standard deviation and has now been corrected here and in table 1.

FIGURE 1
Thank you for including this. Line 261: please hyphenate QoL surveys EQ-5D and SF-36 in the caption
Apologies, this has now been corrected in the figure 1 caption.

FIGURE 2
Thank you for kindly altering the colour schemes of your figures to improve accessibility to the reader with colour blindness. Your caption(s) still refer to your previous colour scheme “...(blue) and without (red)…”. Please amend according to the new scheme and throughout, including supplementary files, where relevant.

Apologies, this has now been corrected in the figure 2 caption.

DISCUSSION
The discussion (and preceding results section) achieves much better clarity regarding the implications of your study outcomes than the abstract/author summary.  I would encourage you to cross reference any specific changes you make to the abstract/author summary with these sections to ensure consistency of language and terms, thus interpretation, throughout the manuscript.

Thank you. We have restructured the abstract to be more consistent with the discussion section.

Line 369: “Ours is the largest study to examine whether…” please temper assertations of primacy – suggest “to our knowledge” or something similar

This sentence has been updated as follows:-

“Ours is the largest study, to our knowledge, to examine whether comorbidity predicts change in quality of life over time or in response to treatment in trial participants [24–29].” Please see page 31, lines 373-374.

LANGUAGE
Please ensure the term disease is replaced with condition or something similar in the title and please check throughout for the same

This has been updated as above.

SOCIAL MEDIA
Please include any twitter handles for your institution/funding body etc such that we can help to extend the reach of your research

We have added the twitter handles for 1) the Public Health Department of the University of Glasgow and 2) Wellcome Trust.
Please see page 2 line 26 and page 4 line 70.

Comments from Reviewers:

Reviewer #1: Thanks for the revised manuscript and responses to my review. Compiling the information into the GitHub repository made completing the re-review much easier.

The sensitivity check with penalised splines is consistent with the polynomial terms, I think this looks fine to me.

I don't think there are any changes needed with the coefficient (my question about interpretability of the beta coefficient), I think you are right that there aren't any benchmarks because it's almost always normally an input in a CE analysis (which has a more obvious interpretation).

The explanation about the priors was helpful thank you - with the additional information available via the appendix I don't think there's any changes that need to be made here.
I happily recommend the manuscript be accepted.

Thank you.

Reviewer #2: Thank you for further reflecting on how to position this paper in the context of research on multimorbidity and comorbidity. I think the additional contributions clarifies the approach taken to conceptualising multimorbidity and justifying the selection of comorbidities.

Thank you.

Reviewer #3: I was pleased to see this revised article.

I must first apply some peer review to the editorial requirements with regard to how systematic reviews should be presented.  To simplistically apply quality standards used for a conventional review to an IPD meta-analysis is simply wrong.  This is an analysis on data are available in an IPD resources.  It is simply not feasible to include trials published within six months.  There is quite clearly no need to update the search to being within the previous six months.  Further I suggest that conventional risk of bias assessment is also inappropriate. The authors are not here trying to suggest any main effects of an intervention. Rather they are looking at within trials data to help us better understand effects of co-morbidity. Above a very low bar for quality, that is met by having data of suitable quality to be included, and evidence of robust randomisation all these data will be of good quality. Essentially the data are what the data are.
I remain very surprised that the authors think it a novel finding that people with multi-morbidity are less well, and have poorer outcomes is a novel finding.  There is a vast amount of observational data, and indeed clinical experience, on this matter. I would also suggest that the highly selective nature of recruitment to the included trials means that these data are far inferior to the existing observational data on this point.  Whilst these data need reporting the really novel finding from this work is that multi-morbidity does not moderate outcome, within this dataset.  Thus I remain surprised that the authors report effect sizes for their first two analyses in the abstract when not including the (non) effect size for treatment moderation.

Thank you for your comments. We have now restructured the abstract to put more emphasis on the finding that comorbidity count at baseline did not alter effect of treatment on quality of life. Please see pages 3-4, lines 28-66.
